# Persistent Gliosis Interferes with Neurogenesis in Organotypic Hippocampal Slice Cultures

**DOI:** 10.3389/fncel.2016.00131

**Published:** 2016-05-18

**Authors:** Johannes Gerlach, Catharina Donkels, Gert Münzner, Carola A. Haas

**Affiliations:** ^1^Department of Neurosurgery, Experimental Epilepsy Research, Medical Center – University of FreiburgFreiburg, Germany; ^2^Faculty of Medicine, University of FreiburgFreiburg, Germany; ^3^Faculty of Biology, University of FreiburgFreiburg, Germany; ^4^BrainLinks-BrainTools, Cluster of Excellence, University of FreiburgFreiburg, Germany

**Keywords:** inflammation, cytokine, Notch signaling, astrocyte, microglia, subgranular zone

## Abstract

Neurogenesis in the adult hippocampus has become an intensively investigated research topic, as it is essential for proper hippocampal function and considered to bear therapeutic potential for the replacement of pathologically lost neurons. On the other hand, neurogenesis itself is frequently affected by CNS insults. To identify processes leading to the disturbance of neurogenesis, we made use of organotypic hippocampal slice cultures (OHSC), which, for unknown reasons, lose their neurogenic potential during cultivation. In the present study, we show by BrdU/Prox1 double-immunostaining that the generation of new granule cells drops by 90% during the first week of cultivation. Monitoring neurogenesis dynamically in OHSC from POMC-eGFP mice, in which immature granule cells are endogenously labeled, revealed a gradual decay of the eGFP signal, reaching 10% of initial values within 7 days of cultivation. Accordingly, reverse transcription quantitative polymerase chain reaction analysis showed the downregulation of the neurogenesis-related genes doublecortin and Hes5, a crucial target of the stem cell-maintaining Notch signaling pathway. In parallel, we demonstrate a strong and long-lasting activation of astrocytes and microglial cells, both, morphologically and on the level of gene expression. Enhancement of astroglial activation by treating OHSC with ciliary neurotrophic factor accelerated the loss of neurogenesis, whereas treatment with indomethacin or an antagonist of the purinergic P2Y12 receptor exhibited potent protective effects on the neurogenic outcome. Therefore, we conclude that OHSC rapidly lose their neurogenic capacity due to persistent inflammatory processes taking place after the slice preparation. As inflammation is also considered to affect neurogenesis in many CNS pathologies, OHSC appear as a useful tool to study this interplay and its molecular basis. Furthermore, we propose that modification of glial activation might bear the therapeutic potential of enabling neurogenesis under neuropathological conditions.

## Introduction

In the adult mammalian brain, the generation of new neurons is restricted to two specialized neurogenic niches: the subventricular zone at the walls of the lateral ventricles and the SGZ of the DG. The SGZ is a thin area underneath the GCL, consisting of only a few cellular rows. Nevertheless, it comprises a broad cellular spectrum including multipotent stem cells and their neuronal progeny as well as glial and endothelial cells, which together form a specialized “microenvironment,” necessary for adult neurogenesis (for review see Ming and Song, 2011).

In the SGZ, neurogenesis starts from multipotent, quiescent type-1 stem cells (radial astrocytes), which by asymmetric cell division give rise to strongly proliferative type-2 (or horizontal) stem cells (Seri et al., 2001; Kempermann et al., 2004). After several cell divisions, these newly generated cells differentiate into neuroblasts and further into granule cells, which finally integrate into the local neuronal network (Jessberger and Kempermann, 2003). SGZ neurogenesis increases network plasticity and has been shown to be required for proper hippocampal function, in particular the generation of spatial memory (Kee et al., 2007; Garthe et al., 2009; Trinchero et al., 2015).

Neurogenesis is not a linear process but rather a sophisticated and fine-tuned sequence of proliferation and differentiation of several types of neuronal precursor cells, which is influenced by a variety of physiological and pathological stimuli (Lugert et al., 2010; Mu et al., 2010; Ming and Song, 2011). Adult neurogenesis has also become a candidate for replacement therapy of neurons lost after CNS insults. However, neurogenesis itself is frequently affected under neuropathological conditions (Mu and Gage, 2011). Therefore, a detailed understanding of the cellular microenvironment and the molecular factors that permit and facilitate adult neurogenesis is important.

Organotypic hippocampal slice cultures tightly reflect the cellular composition as well as connectivity properties of the postnatal hippocampus while lacking the blood-brain barrier. Therefore, OHSC are a suitable tool to study the hippocampal network under well-defined *in vitro* conditions and with the possibility of precise pharmacological intervention (Stoppini et al., 1991; Buchs et al., 1993; Mellentin et al., 2006; Raineteau et al., 2006). In the past, OHSC have been used to analyze diverse physiological and pathological processes reaching from genetic and molecular to synaptic and network studies (Toni et al., 1997; Vlachos et al., 2012; Tinnes et al., 2013; Chai et al., 2014; Pusic et al., 2014; Schneider et al., 2015).

In recent years, hippocampal neurogenesis has become a subject of intense research and, consequentially, was also studied in OHSC (Kamada et al., 2004; Raineteau et al., 2004; Sadgrove et al., 2005, 2006; Bunk et al., 2010; Lee et al., 2012; Perez-Gomez and Tasker, 2012). It was shown that a variety of factors like the application of the glutamate receptor agonists *N*-methyl-D-aspartate (NMDA; Bunk et al., 2010) and kainate (Sadgrove et al., 2005), growth factors (Laskowski et al., 2005), or the addition of serum to the culture medium (Raineteau et al., 2004) strongly influence the *in vitro* generation of new granule cells.

As OHSC contain the whole postnatal DG, which gives rise to the adult SGZ, and in fact do exhibit spontaneous neurogenesis, it is surprising that Namba et al. (2007) were the first to compare this *in vitro* neurogenesis to the *in vivo* equivalent. Immediately after preparation, OHSC exhibit a neurogenesis rate comparable to the *in vivo* condition, but already after 1 week of cultivation neurogenesis strongly decreases. As up to now it is unknown why neurogenesis is lost *in vitro*, we aimed at uncovering the cellular processes and factors interfering with neurogenesis in OHSC.

To address this question, we followed the dynamics of neurogenesis by using OHSC from POMC-eGFP mice, in which the POMC promotor drives the expression of eGFP exclusively in newly generated and immature granule cells (Cowley et al., 2001; Overstreet et al., 2004). Additionally, we analyzed the gene expression profile of OHSC during the course of cultivation. We provide evidence that a strong and long-lasting activation of glial cells takes place in OHSC, which seems to be centrally involved in the loss of neurogenesis, since anti-inflammatory treatments significantly protected neurogenesis.

## Materials and Methods

### Animals

For experiments in which the eGFP signal intensity was measured we used transgenic POMC-eGFP mouse pups. For all other experiments, C57BL/6N mice were used. All animal procedures were carried out in accordance with the guidelines of the European Community’s Council Directive of 22 September 2010 (2010/63/EU) and were approved by the regional council (Regierungspräsidium Freiburg).

### Organotypic Hippocampal Slice Cultures

Organotypic hippocampal slice cultures were prepared using the interface method described by Stoppini et al. (1991). Briefly, P6–P7 pups of C57BL/6N or POMC-eGFP mice were anesthetized by isoflurane and decapitated. The brain was rapidly removed and both hippocampi were dissected in ice-cold preparation medium (75% minimal essential medium, MEM, 25% basal medium Eagle, BME, pH 7.2) and transversally cut (400 μm) using a McIlwain tissue chopper. Intact OHSC from the septal two-thirds of both hippocampi were then transferred onto *Millicell* cell culture inserts (Merck Millipore) and cultivated for 5 days with nutrition medium (46% MEM, 25% BME, 25% heat-inactivated horse serum supplemented with 0.65% glucose and 2 mM glutamine, pH 7.2). After 5 DIV, the medium was changed to a serum-free Neurobasal-A medium containing 2% B-27 supplement, as it was shown that serum affects the *in vitro* neurogenesis (Raineteau et al., 2004). All media and supplements were purchased from Gibco/Thermo Fisher Scientific. The medium was changed every second day.

### Immunohistochemistry

**FIGURE 1:**
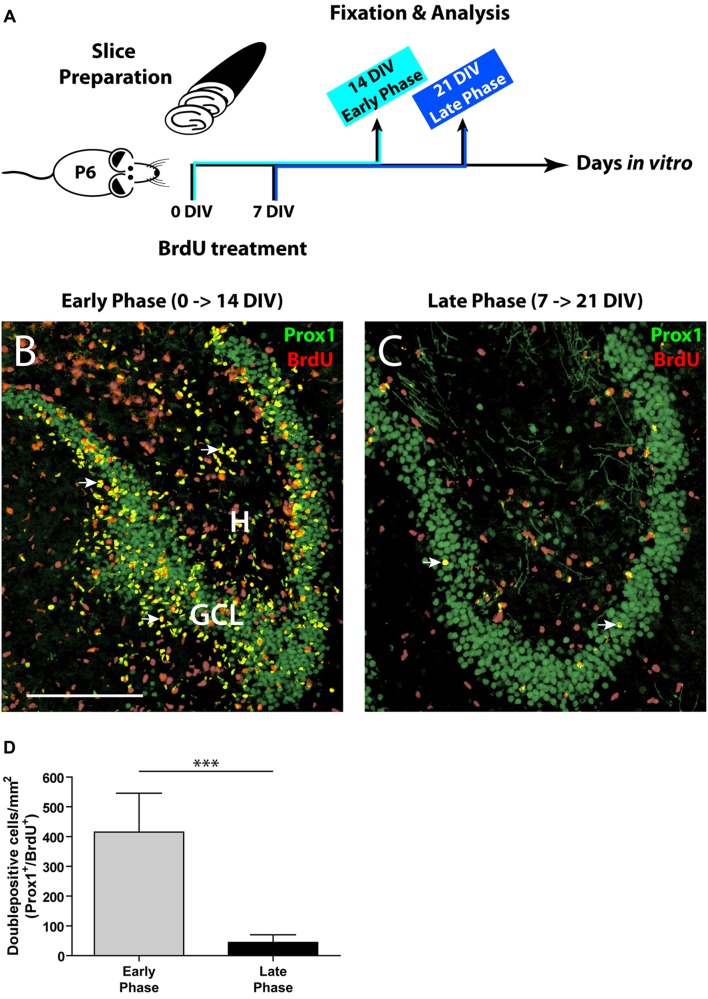
**Comparison of the initial and the late phase of neurogenesis in OHSC. (A)** Time line of the experimental design. OHSC were treated at 0 or 7 DIV with BrdU for 48 h and fixed after an additional incubation period of 14 DIV. **(B,C)** Representative images of the DG area in OHSC cryosections double-immunolabeled for Prox1 (green) and BrdU (red). Double-positive cells (yellow, marked by arrows) are newly born granule cells. H, hilus; GCL, granule cell layer. Scale bar: 200 μm. **(B)** Early phase of OHSC neurogenesis. During the first 48 h after OHSC preparation, many cells were mitotically active and incorporated BrdU (red nuclei). The majority of BrdU-positive cells within the GCL are BrdU/Prox1^+/+^. **(C)** Late phase of OHSC neurogenesis. While, after a cultivation period of 7 days, some cells in the DG area still proliferate, only very few cells are BrdU/Prox1^+/+^. **(D)** Quantification of BrdU/Prox1^+/+^ cells in the DG area. During 1 week of cultivation, neurogenesis has dropped by approximately 90% when compared to the initial value. For sample comparison, an unpaired *t*-test with Welch’s correction was used. Values are displayed as the mean ± SD, *n* = 13, ^∗∗∗^*p* < 0.001.

Organotypic hippocampal slice cultures were fixed in 4% PFA overnight at 4°C and subsequently rinsed several times in 0.1 M PB, pH 7.4. Immunostaining was performed either with whole slices or after cryoprotection of OHSC (25% sucrose, overnight at 4°C) and re-slicing [12 μm (**Figure 5** and Supplementary Figure S1) or 20 μm (**Figure 1**)] on a cryostat (Leica Biosystems). Tissue sections were mounted on glass slides, air-dried, rinsed in 0.1 M PB, and incubated with antibodies against NeuN (MAB377, Merck), prospero homeobox protein 1 (Prox1, Abcam), BrdU (Oxford Biotechnology), activated caspase-3 (Act. Casp-3, R&D Systems), S100β (Swant), GFAP (Santa Cruz Biotechnology), Iba1 (Wako Chemicals), or CD68 (Abcam) using standard immunohistochemical protocols. For whole slice stainings, incubation times of the primary and secondary antibodies were extended to 24 h at room temperature. Detection of the first antibody was performed by using Cy2- or Cy3-conjugated secondary antibodies (Jackson ImmunoResearch Laboratories). Sections were counterstained with DAPI, coverslipped in fluorescence mounting medium (Dako) and analyzed using an epifluorescence (Axio Imager 2, Carl Zeiss) or confocal microscope (Olympus FluoView FV10i).

To label proliferating cells, OHSC were treated with 10 μM BrdU for 48 h directly after the slice preparation (0 DIV) or after 7 DIV. To assess the rate of neurogenesis, BrdU-treated OHSC were kept in culture for additional 14 DIV, fixed, and double-immunostained with antibodies against BrdU and Prox1. To permit antigen recognition by the anti-BrdU antibody, sections were incubated in 2 N HCl for 30 min at 37°C, and subsequently the pH was normalized by rinsing the sections in Tris-buffered saline (TBS), pH 8.5, and in 0.1 M PB prior to the pre-incubation step.

### Quantification of *In Vitro* Generated Granule Cells

We considered cells which had incorporated BrdU and subsequently differentiated into Prox1- positive granule cells within 14 DIV as *in vitro* generated neurons. To determine the density of newly generated granule cells in OHSC, all Prox1/BrdU double-immunolabeled cells were counted per section, and this value was then divided by the DG area (cells per mm^2^). Cell counting was performed with ImageJ software, and statistical analysis was done with GraphPad Prism 4 software. For sample comparison of BrdU/Prox1^+/+^ cells, an unpaired *t*-test with Welch’s correction was used. Significance level was set at *p* < 0.05.

### POMC-eGFP Signal Quantification

The eGFP signal intensity in the DG was quantified in living OHSC from POMC-eGFP mice and used as an estimate for the population size of immature granule cells and neuronal progenitors, reflecting the overall OHSC neurogenesis. To quantify eGFP fluorescence intensity during the whole cultivation period, photomicrographs of the DG of every OHSC were taken daily with constant exposure time settings (2–4 photos per OHSC, each with 1 s exposure time, ISO 400, and constant white balance), using a digital SLR camera (Olympus E-450) attached to a fluorescence microscope (Olympus CKX41 with U-RFL-T). Images were converted to grayscale, and the eGFP signal intensity was quantified as integrated density using ImageJ software. Values were corrected by background subtraction: integrated density – (measured area × mean background signal). The mean background signal was calculated for each experiment. Background signals were measured in areas without eGFP signal and from several OHSC. To test for potential bleaching effects, POMC-eGFP OHSC were cultivated without any fluorescence light exposure and fixed after different cultivation periods. Subsequently, these OHSC were cryosectioned (12 μm), photomicrographs were taken using an epifluorescence microscope and constant exposure times, and the eGFP fluorescence intensity was quantified. Statistical analysis was performed with GraphPad Prism 4 software. For sample comparison, one-way ANOVA with Tukey’s post-test was used. Significance level was set at *p* < 0.05.

### Quantification of Resting and Activated Microglia

In order to quantify the degree of microglia activation, we performed double immunolabeling for Iba1 and CD68 (a marker for activated microglia cells) in cryosections of OHSC. Using Image J, we counted all cells/section which were Iba1-positive but CD68-negative (resting microglia) and all cells double-positive for Iba1 and CD68 (activated microglia). These values were then divided by the area of the whole OHSC (cells per mm^2^). Statistical analysis was performed with GraphPad Prism 4 software. For sample comparison of resting versus activated microglia, a two-way ANOVA with Bonferroni post-tests was used, while sample comparison within each group was done using a one-way ANOVA with Tukey’s post-test. Significance level was set at *p* < 0.05.

### Pro- and Anti-Inflammatory Treatments

To induce and accelerate astroglial activation, OHSC were treated for the first two DIV with CNTF (20 ng/ml, Cell Concepts GmbH), a potent inducer of reactive gliosis (Levison et al., 1996). Afterwards, the medium was changed back to the normal nutrition medium. To counteract inflammatory processes, we treated OHSC either with a specific antagonist of the purinergic P2Y12 receptor (PSB 0739, 100 nM, Tocris Bioscience) or with indomethacin (50 μM, Sigma-Aldrich GmbH) during the entire cultivation period.

### Gene Expression Analysis (RT-qPCR)

For RNA preparation, 4–6 OHSC from different mouse pups were pooled (= one sample) and incubated in 300 μl RNA later (Quiagen). Total RNA was extracted and purified using the RNeasy Mini Kit (Quiagen) and reversely transcribed into cDNA with the Maxima First Strand cDNA synthesis Kit (Thermo Fischer Scientific). cDNA was diluted 1:50 in sterile water, and reverse transcription quantitative polymerase chain reaction (RT-qPCR) was performed using an iQ5 Real-Time PCR Detection System (Bio-Rad Laboratories GmbH) in the presence of SYBR Green (Thermo Fischer Scientific), as described previously (Tinnes et al., 2011). For calculation of relative mRNA expression rates, the Cy0 method was applied as a tool for accurate and precise quantification (Guescini et al., 2008). Target gene expression was normalized to ribosomal protein S12 (expressed as ΔCt). For every transcript, the mean relative gene expression at 0 DIV was defined as 100%. Statistical analysis was performed with GraphPad Prism 4 software. For sample comparison, one-way ANOVA with Tukey’s post-test was used. Significance level was set at *p* < 0.05.

## Results

### OHSC Rapidly Lose Their Neurogenic Capacity

As a first step, we determined the proliferative activity in OHSC. We applied BrdU to the OHSC either immediately after the slice preparation (0 DIV, “early phase”) or after 1 week of cultivation (7 DIV, “late phase,” **Figure 1A**), since according to Namba et al. (2007) neurogenesis decreases mainly during the first week of cultivation. To monitor the differentiation of BrdU-positive cells, we cultivated the BrdU-treated slices for additional 14 DIV and performed double immunostainings for BrdU and Prox1, a marker of granule cells and their progeny (Liu et al., 2000; Pleasure et al., 2000). Cells double-positive for BrdU and Prox1 (BrdU/Prox1^+/+^) represent newly generated granule cells and served as a marker for ongoing neurogenesis.

In the early phase of cultivation, OHSC showed a high number of BrdU-positive cells, localized inside and outside the GCL (**Figure 1B**). Double labeling with antibodies against GFAP or Iba1 revealed that most BrdU^+^ cells outside the GCL were proliferating glial cells (data not shown), while inside the GCL most cells were BrdU/Prox1^+/+^ (**Figure 1B**). In contrast, OHSC showed a strongly reduced number of BrdU/Prox1^+/+^ cells during the late phase of cultivation (by approximately 90%) when compared to the early phase (**Figures 1B–D**) indicating a significant decline of neurogenesis.

**FIGURE 2:**
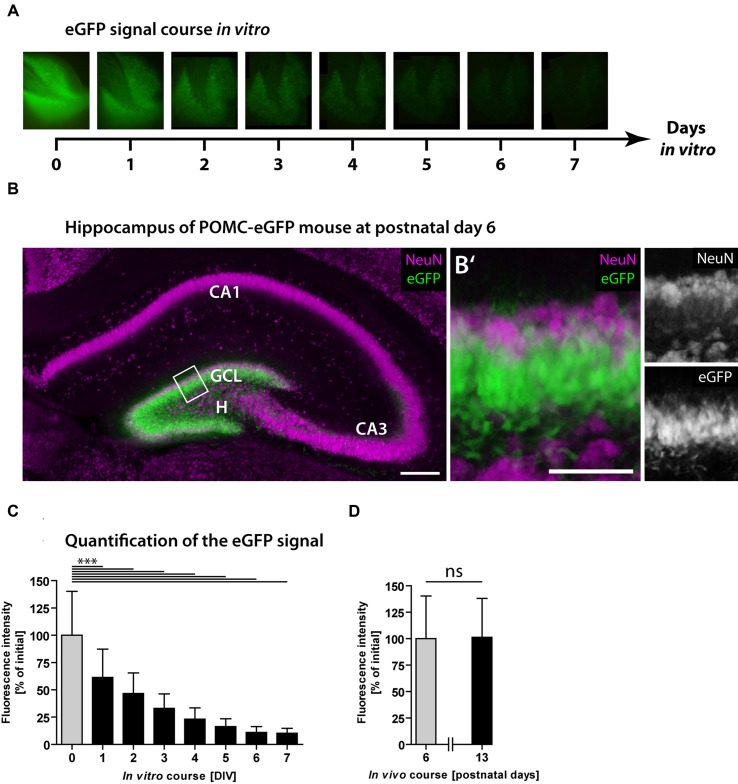
**Loss of neurogenesis in OHSC from POMC-eGFP reporter mice**. The eGFP signal intensity was measured daily in OHSC during 7 DIV and served as a marker for neurogenesis. **(A)** Time scale and representative photomicrographs showing the eGFP signal of the DG in one OHSC over a period of 7 DIV. Pictures were taken with constant exposure times. **(B)** Immunostaining for NeuN (purple) shows neuronal cell layers in the hippocampus of a POMC-eGFP mouse at P6, the time point of OHSC preparation. GCL, granule cell layer; H, hilus. Scale bar: 200 μm. **(B′)** Higher magnification of the suprapyramidal blade of the DG (shown in **B**) depicts the GCL and part of the hilus. At P6, the majority of immature granule cells express eGFP but not NeuN. Scale bar: 50 μm. **(C,D)** Quantification of the eGFP signal intensity *in vitro* during 7 DIV **(C)** and *in vivo*, in freshly prepared and non-cultivated OHSC at P6 and P13 **(D)**. Note the decline in eGFP signal intensity during the OHSC cultivation period indicating a fast and strong reduction of neurogenesis **(C)**, which is not observed during physiological postnatal development **(D)**. For sample comparison, one-way ANOVA with Tukey’s post-test was used. Values are displayed as the mean ± SD (P6, 0–7 DIV, *n* = 9) and (P13, *n* = 3). ^∗∗∗^*p* < 0.001, ns *p* ≥ 0.05.

Next, we used POMC-eGFP mice to monitor the neurogenic rate dynamically over time. As eGFP is expressed exclusively in immature granule cells, the fluorescence intensity reflects the population size of newborn neurons and was used to estimate the degree of neurogenesis. The eGFP signal intensity of every OHSC was measured daily over a period of 7 DIV (**Figure 2A**). At the time point of OHSC preparation (0 DIV), the majority of granule cells was eGFP-positive but NeuN-negative (**Figures 2B,B**′). NeuN is a marker of mature neurons (Mullen et al., 1992) and at this developmental time point exclusively stains granule cells in the outer GCL, while eGFP-positive cells are located in the inner to mid portion of the GCL. While at 0 DIV eGFP was strongly expressed by the majority of granule cells (**Figures 2A,B**), from 1 to 7 DIV the eGFP signal intensity gradually decreased and finally reached 10% of the initial value (**Figures 2A,C**). These results point to a fast shrinkage of the population size of young granule cells in OHSC and confirm the drop of neurogenesis shown by the BrdU/Prox1 double-immunostainings (**Figure 1**). As the observed eGFP signal decline could be due to normal postnatal maturation of young, eGFP-positive granule cells, we additionally analyzed the respective course of the eGFP signal *in vivo*. For this, we measured the eGFP signal intensity in freshly prepared hippocampal slices at P6 and P13, which are equivalent time points to cultivated OHSC at 0 and 7 DIV, respectively. In contrast to the strong decrease seen during OHSC cultivation, the physiological postnatal development did not cause a reduction of the eGFP signal intensity *in vivo* (**Figure 2D**).

We also tested whether bleaching of the fluorescence, caused by daily light exposure, might be responsible for the observed decay of the eGFP signal. For this, we cultivated OHSC without any fluorescence light exposure, fixed them after different cultivation periods, and measured the eGFP fluorescence intensity in photomicrographs (Supplementary Figure S1). Even without daily light exposure, OHSC exhibited a strong and gradual decay of the eGFP signal, which finally reached 20% of the initial value. These experiments revealed that neither physiological, postnatal granule cell maturation nor fluorescence fading is responsible for the observed eGFP signal loss during OHSC cultivation.

**FIGURE 3:**
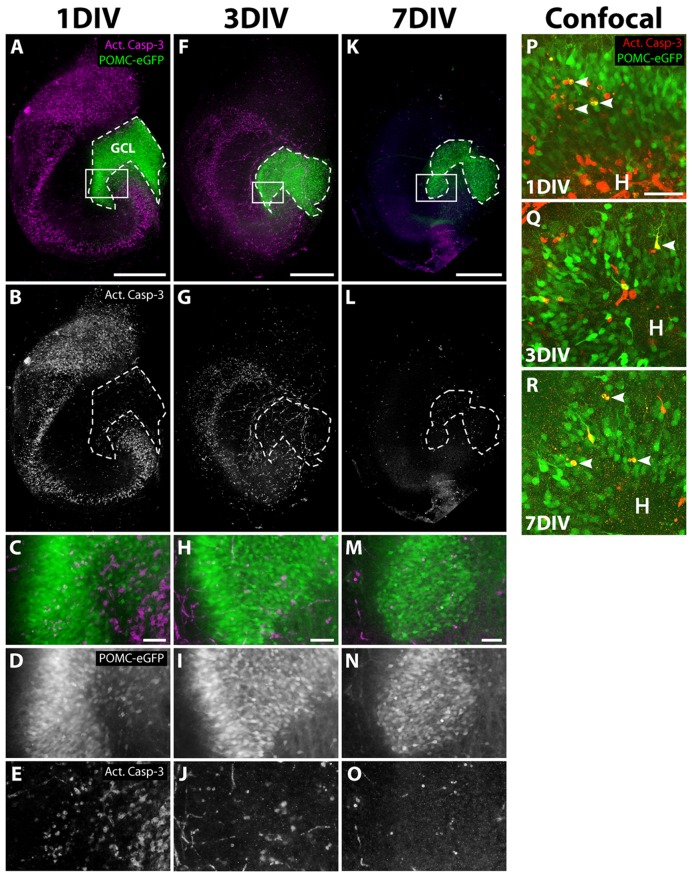
**Apoptosis during OHSC cultivation**. OHSC from POMC-eGFP mice were cultivated for 1, 3, or 7 DIV, fixed, and stained as whole slices with an antibody against activated caspase-3 (Act. Casp-3). **(A–O)** Photomicrographs, obtained by an epifluorescence microscope, reveal many apoptotic cells in all neuronal cell layers except for the GCL during the first days of cultivation. **(C–E,H–J,M–O)** High power views of the DG, marked by white frames in **(A,F,K)**. At 1 DIV, the extent of apoptosis is highest **(A–E)**, followed by a subsequent decline at 3 DIV **(F–J)** and at 7 DIV **(K–O)**. Scale bar: 500 μm (whole slice images: **A,F,K**) and 50 μm (magnifications: **C,H,M**). Please note that the eGFP photomicrographs were taken with increasing exposure times (1 DIV: 950 ms, 3 DIV: 1600 ms, 7 DIV: 5000 ms). **(P–R)** Confocal mircoscopical analysis confirms the low apoptotic rates of granule cell progenitors at 1, 3, and 7 DIV. At 1 and 3 DIV, when high rates of cell death are visible, most apoptotic cells within the DG are eGFP-negative. Images are Z projections of 10 stacks (1 μm step size). Scale bar: 50 μm.

To rule out that the decline of the eGFP signal was caused by degeneration of the progenitor cell population, we performed immunostaining for activated caspase-3 (Act. Casp-3), an apoptosis marker (Nicholson et al., 1995; Porter and Janicke, 1999), during the cultivation of OHSC from POMC-eGFP mice (**Figure 3**). At 1 DIV, many Act. Casp-3-positive cells were detectable in all neuronal cell layers except for the GCL, in which only a few cells were apoptotic (**Figures 3A–E**). At later time points, the cell death rate decreased, and at 7 DIV only a few apoptotic cells were present throughout the whole OHSC (**Figures 3F–O**). We used confocal microscopy to analyze in detail whether there was degeneration of eGFP-positive cells (**Figures 3P–R**). We found only single apoptotic granule cell progenitors at any of the time points investigated.

Together, our data indicate that the observed loss of neurogenesis is not caused by degeneration of the respective granule cell progenitors.

### Neurogenesis-Related Genes Are Down-Regulated during Cultivation

**FIGURE 4:**
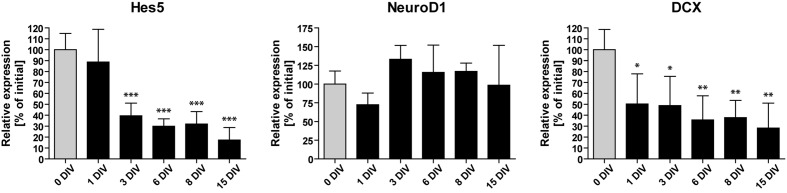
**Time course of neurogenesis-related gene expression**. RT-qPCR analyses of Hes5, NeuroD1, and DCX mRNA *in vitro*. Initial values (0 DIV) are set as 100% and are shown as gray columns. The expression of Hes5 and DCX mRNA is rapidly downregulated, while NeuroD1 mRNA expression remains relatively constant during 15 DIV. For sample comparison, one-way ANOVA with Tukey’s post-test was used. Values are displayed as the mean ± SD. *n* = 3–5 samples per time point (see Materials and Methods). ^∗^*p* < 0.05, ^∗∗^*p* < 0.01, ^∗∗∗^*p* < 0.001.

To identify possible factors involved in the observed decline of neurogenesis, we performed RT-qPCR analyses of OHSC at different time points during the cultivation period. We measured the relative gene expression levels of Hes5, NeuroD1, and DCX (**Figure 4**). Hes5 is a transcription factor, the expression of which is regulated by the Notch signaling pathway (Bertrand et al., 2002; Ross et al., 2003). Notch signaling is essential for stem cell maintenance in the neurogenic niche and its loss was shown to cause stem cell depletion by inducing neuronal differentiation (Imayoshi et al., 2010; Sibbe et al., 2012). NeuroD1, on the other hand, is a pro-neurogenic transcription factor, known to be essential for granule cell differentiation and maturation (Lee et al., 1995; Liu et al., 2000; Pleasure et al., 2000; Gaudillière et al., 2004). DCX is a microtubule-associated protein, exclusively expressed in the hippocampus by immature dentate granule cells (Francis et al., 1999; Brown et al., 2003; Couillard-Despres et al., 2005).

We found that the expression of NeuroD1 remained relatively stable during OHSC cultivation. In contrast, Hes5 as well as DCX expression were rapidly down-regulated within the first 3 DIV to 40 and 49% of their initial expression levels, respectively. Subsequently, the expression of Hes5 and DCX further decreased until it reached ∼20–30% of the initial expression values. Taken together, these results confirm the fast and long-lasting disturbance of the neurogenic niche in OHSC, as described above (**Figures 1** and **2**).

### Occurrence of a Fast and Strong Activation of Astro- and Microglial Cells

**FIGURE 5:**
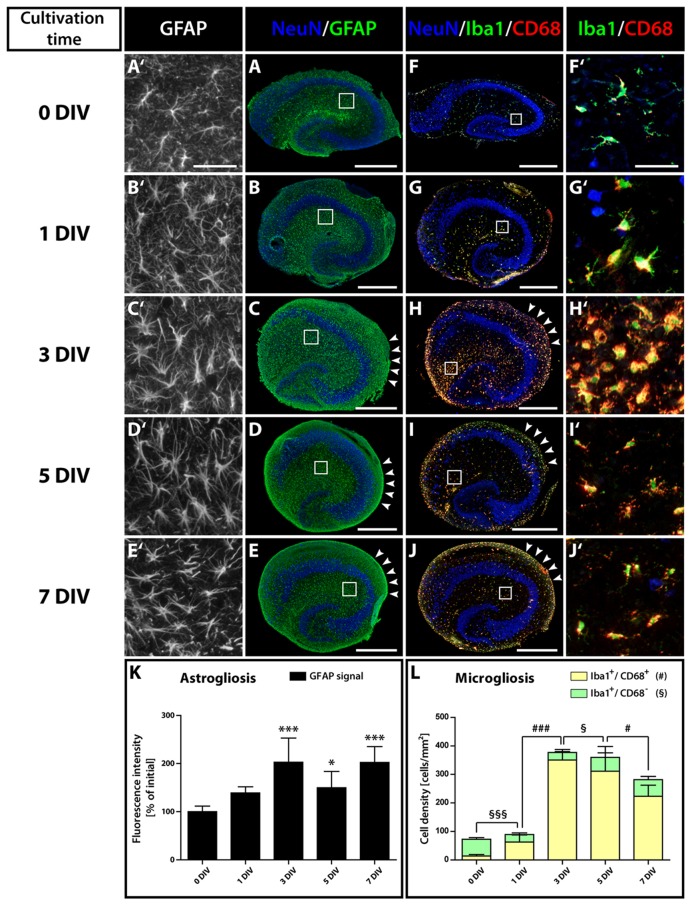
**Time course of astro- and microgliosis in OHSC. (A–E′)** Double-immunolabeling for GFAP (green, astrocytes) and NeuN (blue, neurons). **(F–J′)** Double-immunolabeling for Iba1 (green, microglia), CD68 (red, activated microglia), and NeuN (blue). Photomicrographs show cryosections of OHSC **(A–J)** or respective magnifications from regions of the hippocampal fissure **(A′–J′)**, highlighting the morphological characteristics of both cell types. Initially (0 DIV), astrocytes appear as small, stellate-shaped cells with thin and rather short GFAP-positive processes **(A′)**. At later time points, astrocytes display an increased soma size and become strongly GFAP-positive **(B′,C′)**. Microglia also possess small somata with thin and highly ramified cell processes at 0 DIV **(F′)**, but already at 1 DIV enlarged somata and CD68 immunoreactivity can be observed **(G′)**. From 1 to 3 DIV the cell density of activated microglia strongly increases **(F′–H′)**. Scale bar: 500 μm (whole OHSC, **A–J**) and 50 μm (magnifications, **A′–J′**). Quantification of GFAP immunoreactivity **(K)** and of the density of resting and activated microglia **(L)** reveals a fast and long-lasting activation of both cell types. For sample comparison, one-way ANOVA with Tukey’s post-test was used, while for sample comparison of resting versus activated microglia a two-way ANOVA with Bonferroni post-tests was used. Values are displayed as the mean ± SD. *n* = 5; ^∗^*p* < 0.05, ^∗∗∗^*p* < 0.001, non-activated microglia: §*p* < 0.05, §§§*p* < 0.001, activated microglia: #*p* < 0.05, ###*p* < 0.001.

To elucidate changes in the microenvironment of the neurogenic niche potentially accounting for the loss of neurogenesis, we studied the degree of glial cell activation in OHSC. It is known that inflammatory conditions can have a deleterious influence on adult hippocampal neurogenesis *in vivo* (Belarbi and Rosi, 2013). Immunostainings for GFAP, Iba1, and CD68 at different cultivation time points revealed a strong and long-lasting activation of astrocytes and microglia in OHSC (**Figure 5**). While initially (0 DIV) both cell types exhibited a resting phenotype with highly ramified processes and small somata (**Figures 5A′,F′**), they displayed an increased soma size already after 1 DIV (**Figures 5B′,G′**). Especially microglial cells turned into an activated, amoeboid phenotype, strongly expressing CD68 (**Figures 5G′,H′**). We quantified the extent of astrogliosis by measuring the GFAP immunoreactivity (**Figure 5K**). In fact, GFAP immunoreactivity revealed a highly significant increase between 0 and 3 DIV and quite constant levels from 3 to 7 DIV. While astrocytes in the center of OHSC did not show additional activation after 3 DIV, they remained activated at the OHSC surface, forming the well-known glial scar (**Figures 5C–E**, arrowheads).

Microgliosis was even more pronounced. Counting activated (Iba1^+^/CD68^+^) microglial cells (**Figure 5L**) revealed an increase of 4.5-fold at 1 DIV and of 25-fold at 3 DIV. While at 0 DIV only 19% of Iba1-positive microglia expressed CD68, at 1 DIV 70% and at 3 DIV 93% of the microglial cells co-localized with CD68. As the overall number of microglial cells dramatically increased from 1 to 3 DIV, microglia are likely to proliferate within this time window, whereas the initial activation during the first day (from 0 to 1 DIV) was not accompanied by a significant increase in cell numbers. Despite the fact that the density and soma size of microglia seemed to decrease in the center of OHSCs after 3 DIV (**Figures 5H′–J′**), our quantification revealed, however, that the overall density of activated microglia was only slightly reduced (**Figure 5L**). After 7 DIV, 79% of the microglia still expressed CD68. Similar to astrogliosis, microglia activation was maintained on the surface of OHSC (**Figures 5H–J**, arrowheads).

**FIGURE 6:**
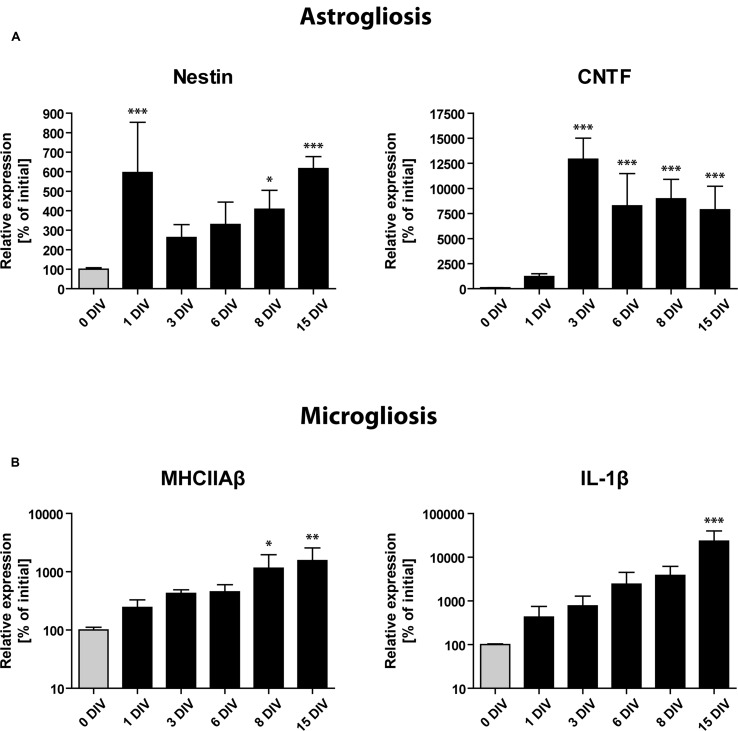
**Gene expression profile of glial activation markers during OHSC cultivation by RT-qPCR analysis**. Initial expression values (0 DIV) are set as 100% and shown as gray columns. **(A)** Gene expression of markers for astrogliosis (Nestin, CNTF) points to a very fast and strong activation of astrocytes, reaching its maximum within 1–3 DIV. **(B)** Gene expression of microglial activation markers (MHCIIAβ, IL-1β). Microgliosis follows a slower but exponential increase dynamic, which does not seem to reach a plateau during 15 DIV. For sample comparison, one-way ANOVA with Tukey’s post-test was used. Values are displayed as the mean ± SD. *n* = 3–5 samples per time point (see Materials and Methods). ^∗^*p* < 0.05, ^∗∗^*p* < 0.01, ^∗∗∗^*p* < 0.001.

To complement our immunohistochemical findings, we performed RT-qPCR analysis of transcripts known to be specifically expressed by activated astrocytes and microglial cells (**Figure 6**): (1) The intermediate filament protein Nestin, a frequently used marker of neuronal stem cells within the SGZ, has been shown to be strongly expressed by activated astrocytes during inflammatory conditions (Frisén et al., 1995), (2) CNTF mediates the activation of astrocytes and was also shown to be expressed by activated astrocytes (Ip et al., 1993; Levison et al., 1996; Escartin et al., 2006), (3) and (4) As markers for microglia activation, we used MHCIIAβ and the pro-inflammatory cytokine interleukin 1β (IL-1β; Graeber et al., 2011; Kettenmann et al., 2011).

RT-qPCR analysis revealed a markedly enhanced expression of all above mentioned markers of gliosis during the time of OHSC cultivation (**Figure 6**). Already at 1 DIV, we found the expression of Nestin mRNA to be increased more than fivefold, followed by a short drop until 3 DIV and a consecutive rise until 15 DIV (**Figure 6A**). CNTF mRNA showed a slower but stronger induction with expression levels being 100-fold increased after 3 DIV when compared to non-cultured controls (**Figure 6A**). Microglia-specific transcripts showed a slower but exponential increase (**Figure 6B**). MHCIIAβ expression was elevated more than 10-fold after 15 DIV, while IL-1β reached levels that were more than 100-fold higher than initial values (**Figure 6B**). These results confirm that a strong astro- and microgliosis takes place in OHSC during cultivation with slightly different dynamics.

It was recently shown that under strong epileptic conditions neural stem cells start to divide symmetrically and transform into reactive astrocytes (Heinrich et al., 2006; Ledergerber et al., 2006; Sierra et al., 2015). Therefore, the observed astrogliosis in OHSC could also be the consequence of an impaired neurogenesis. To answer this question, we investigated whether the *de novo* generation of astrocytes was restricted to the DG, which would point to the neurogenic niche as a cellular source for reactive astrocytes (Supplementary Figure S2). For this, we treated OHSC at 0, 3, or 7 DIV with BrdU and waited for additional 7 DIV, until we stained them as whole slices with antibodies against BrdU and S100β, a marker of post-mitotic astrocytes. When we analyzed the appearance of *de novo* generated astrocytes within the DG in comparison to the CA1 region, we found that astrocytes originate from proliferating cells in both areas at all time points. This observation shows that reactive gliosis takes place throughout the whole OHSC independently of the presence of a neurogenic niche, wherefore an impaired neurogenesis is unlikely to cause the astrogliosis.

### Anti-inflammatory Treatments Protect Neurogenesis

**FIGURE 7:**
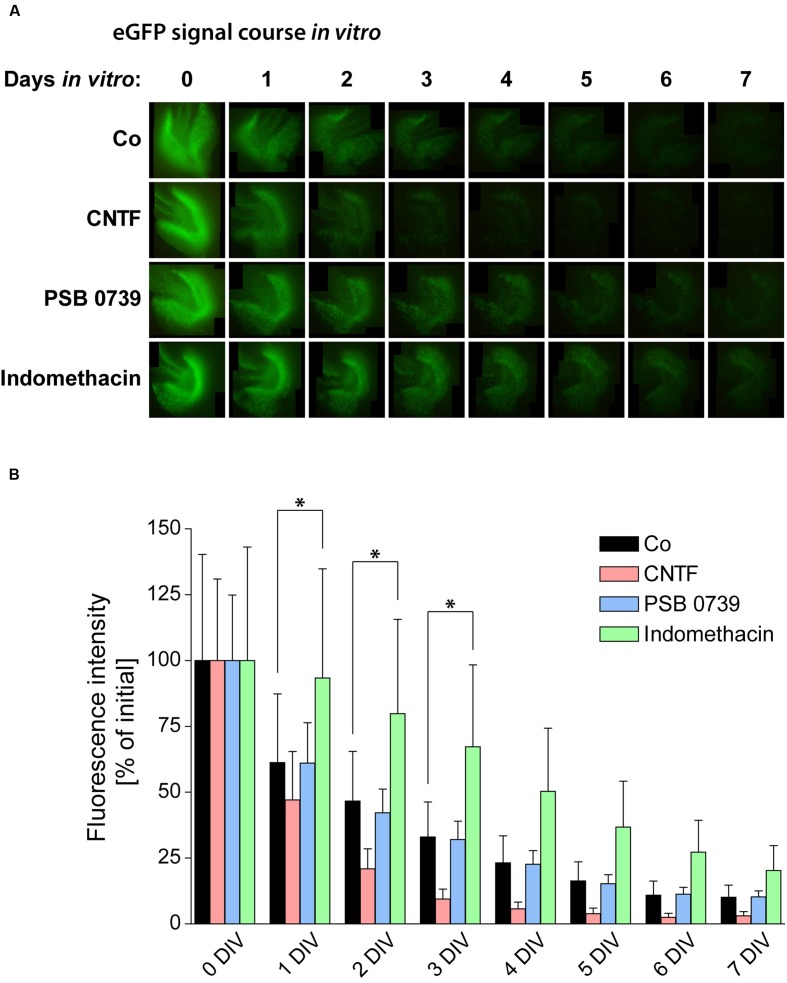
**Pro- and anti-inflammatory treatments and their respective influence on neurogenesis. (A)** Time course of the POMC-eGFP signal in representative OHSC, treated with substances promoting (CNTF) or counteracting (PSB 0739 and indomethacin) glial activation. **(B)** Quantification of the eGFP signal intensity during the cultivation period reveals significant differences between the respective treatments. OHSC were treated with CNTF (20 ng/ml) for 48 h (0–2 DIV) or with PSB 0739 (100 nM) or indomethacin (50 μM) for the entire cultivation period. Enhancing astrogliosis by CNTF treatment accelerates the decay of neurogenesis, while the anti-inflammatory treatment with indomethacin exerts potent protective effects on neurogenesis. PSB 0739 does not alter the course of the eGFP signal intensity. For sample comparison, one-way ANOVA with Tukey’s post-test was used. Values are displayed as mean ± SD. *n* = 9; ^∗^*p* < 0.05.

**FIGURE 8:**
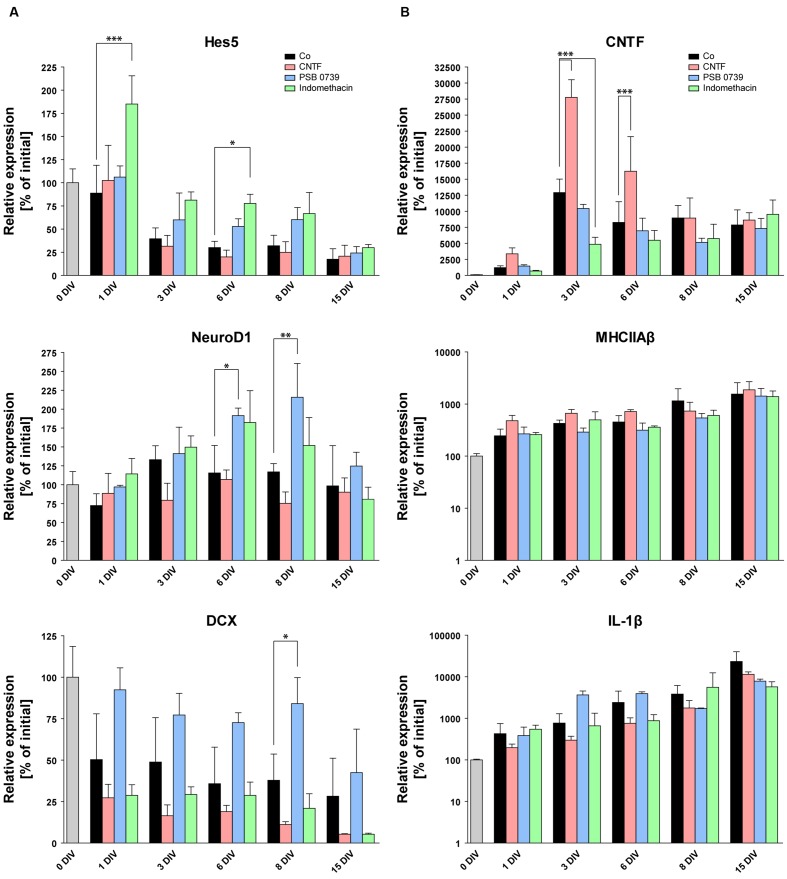
**Pro- and anti-inflammatory treatments and their respective influence on the gene expression profile**. RT-qPCR of markers for neurogenesis **(A)** and glial activation **(B)**. Initial expression values (0 DIV) are set as 100% and shown as a gray columns. While CNTF treatment increases the expression of astrogliosis markers (CNTF, **B**) and causes the downregulation of DCX **(A)**, anti-inflammatory treatments with PSB 0739 and indomethacin significantly increase the expression of markers for neurogenesis (Hes5, NeuroD1, DCX), pointing to an efficient protection of OHSC neurogenesis. On the other hand, PSB 0739 and indomethacin only decreased the expression of CNTF at four of five measured time points, whereas MHCIIAβ and IL-1β expressions were mostly unaffected. For sample comparison, one-way ANOVA with Tukey’s post-test was used. Values are displayed as mean ± SD. *n* = 3–6 samples per time point (see Materials and Methods). ^∗^*p* < 0.05, ^∗∗^*p* < 0.01, ^∗∗∗^*p* < 0.001.

As the loss of neurogenesis is accompanied by a strong and long-lasting activation of glial cells in OHSC, we aimed at counteracting gliosis by the treatment with anti-inflammatory pharmacological substances. To analyze the respective influence on neurogenesis, we again quantified the eGFP signal intensity in OHSC from POMC-eGFP mice (**Figure 7**). First, we tested the effect of an accelerated and enhanced astrogliosis by applying CNTF to the culture medium for the first 48 h of cultivation. CNTF treatment accelerated the decrease of the eGFP signal, pointing to a faster loss of neurogenesis (**Figure 7**). While at 2 DIV the eGFP signal intensity of control OHSC was found to be decreased by 47% in comparison to values at 0 DIV, CNTF-treated OHSC exhibited a decrease of the eGFP signal by 79% (**Figure 7B**). Additionally, we analyzed the gene expression profile by RT-qPCR (**Figure 8**). The anti-neurogenic effects of CNTF were confirmed on the level of DCX gene expression (**Figure 8A**). In CNTF-treated OHSC, DCX expression was decreased at all five analyzed cultivation time points (by 62% on average) when compared to time-matched controls. In contrast, CNTF did not seem to affect the expression levels of Hes5 and NeuroD1 (**Figure 8A**).

We also quantified CNTF gene expression to test whether the CNTF treatment indeed enhanced astroglial activation (**Figure 8B**). Already at 1 DIV, CNTF expression was found to be 2.8-fold higher than in time-matched control OHSC (controls: 1213% ± 280%, CNTF-treated: 3356% ± 943%). After 3 and 6 DIV, the CNTF expression was still increased twofold in CNTF-treated OHSC, followed by a subsequent decline to control levels at 8 and 15 DIV. These results confirm that the CNTF treatment accelerated and enhanced astroglial activation and point to an anti-neurogenic effect. Concerning microgliosis, CNTF treatment had no effect on MHCIIAβ expression but decreased IL-1β expression at all five measured time points (by 58% on average, **Figure 8B**). This result points to an exclusive effect of CNTF onto astroglial activation without increasing microgliosis or the expression of pro-inflammatory cytokines.

To counteract glial activation, we applied (1) a specific antagonist (PSB 0739) of the purinergic P2Y12 receptor, which is known to be centrally involved in the first steps of microglial activation after CNS tissue damage (Haynes et al., 2006), and (2) indomethacin, a non-steroidal cyclooxygenase 1 and 2 inhibitor with anti-inflammatory function (Ji et al., 2013).

PSB 0739 treatment had no effect on the eGFP signal decay in OHSC from POMC-eGFP mice (**Figure 7**). In contrast, indomethacin exerted strongly protective effects: while control OHSC lost 50% of their initial eGFP signal intensities within 2 DIV, indomethacin-treated OHSC reached these levels not before 4 DIV. At 1 and 2 DIV, eGFP levels in indomethacin-treated OHSC were 50–70% higher than in time-matched control OHSC, and from 3 to 7 DIV the eGFP signal was even elevated by 100% above control (**Figure 7**).

To complement these data on the molecular level, we performed RT-qPCR analysis to analyze potential effects of indomethacin and PSB 0739 on the expression of neurogenesis- and gliosis-related genes. The expression of Hes5, which was rapidly reduced in non-treated and in CNTF-treated OHSC, showed stabilized or even increased values at all measured time points with both anti-inflammatory substances (**Figure 8A**). At 1 DIV, Hes5 expression was mostly unchanged in control, CNTF-, and PSB 0739-treated OHSC when compared to values at 0 DIV, while indomethacin-treated OHSC exhibited expression values which were increased by 85%. Until 3 DIV, Hes5 expression decreased in control and CNTF-treated OHSC by 60 and 68%, respectively. In contrast, PSB 0739- and indomethacin-treated OHSC exhibited a reduction of Hes5 expression by only 40 and 19% of their initial values, respectively (**Figure 8A**). Indomethacin exerted stronger effects than PSB 0739 and significantly increased Hes5 expression in comparison to controls at 1 and 6 DIV.

NeuroD1 expression was also increased after both anti-inflammatory treatments, but only PSB 0739 significantly increased the expression levels at 6 DIV (by 66%) and 8 DIV (by 85%; **Figure 8A**). The same was true for the expression of DCX, which was stabilized for up to 8 DIV exclusively by PSB 0739 treatment (84% of initial values versus 38% in controls), whereas indomethacin-treated OHSC exhibited even reduced expression levels (**Figure 8A**). Taken together, these results highlight the beneficial outcome of anti-inflammatory treatments on neurogenesis and confirm the detrimental effect of a strong gliosis developing during the cultivation of OHSC.

As last step, we tested whether PSB 0739 and indomethacin were able to reduce the expression of glia activation markers (**Figure 8B**). Interestingly, neither PSB 0739 nor indomethacin was sufficient to reduce the expression of MHCIIAβ and IL-1β (**Figure 8B**). There were no significant differences or trends visible. However, both anti-inflammatory treatments had an impact on the CNTF expression (**Figure 8B**), which was decreased at four of five time points after PSB 0739 and indomethacin treatment. At 1 and 3 DIV, CNTF expression was down-regulated by 44 and 62% in indomethacin-treated OHSC, respectively (**Figure 8B**). Taken together, we found that PSB 0739 and indomethacin were effective in reducing astro- but not microglia activation.

## Discussion

In the present study, we provide evidence that in OHSC neurogenesis is gradually lost, a process which is accompanied by a fast and strong activation of glial cells. Modifying the degree of glial cell activation by treating OHSC with CNTF, PSB 0739, or indomethacin determined the neurogenic outcome. As anti-inflammatory treatments protected the *in vitro* neurogenesis, the activation of glial cells is likely to be a main reason for the loss of neurogenesis in OHSC.

### OHSC Gradually Lose Their Neurogenic Potential

We showed by quantitative evaluation of BrdU/Prox1 double-immunolabeled cells that the generation of new granule cells *in vitro* decreases by approximately 90% during the first week of cultivation. These results are perfectly in line with the findings of Namba et al. (2007), who reported a drop of neurogenesis by approximately 80% using the same examination time points. Daily measurements of the eGFP signal intensity in OHSC from POMC-eGFP mice confirmed the loss of neurogenesis and revealed a gradual decay over 7 DIV. In accordance with the quantifications of BrdU/Prox1^+/+^ cells, after 7 DIV OHSC exhibited eGFP values that were reduced by 90% when compared to the initial values at 0 DIV.

This gradual decay was neither found to be due to fluorescence fading nor to normal postnatal maturation. Therefore, we asked which cellular processes could underlie the loss of neurogenesis in OHSC. One explanation could be that granule cell progenitors undergo apoptosis due to insufficient synaptic integration, which is a necessary prerequisite for the survival of these cells (Ge et al., 2008; Ming and Song, 2011). The disconnection of the hippocampal network from other brain structures during the OHSC preparation process supports this hypothesis.

However, when we monitored apoptosis with an antibody against activated caspase-3 during OHSC cultivation, we did not find a considerable degeneration of eGFP-positive granule cells. Even after 1 DIV, a time point at which we detected many apoptotic cells in other hippocampal cell layers, we found only few degenerating neurons within the GCL. Confocal laser microscopy confirmed the low number of apoptotic granule cell progenitors at all investigated time points.

The survival of newly born granule cells is also supported by our observation that a notable number of initially labeled, proliferating cells survived and successfully differentiated into mature, Prox1-positive granule cells. Furthermore, NeuroD1, which promotes granule cell differentiation, exhibited a stable expression pattern during OHSC cultivation. Accordingly, studies of other groups had also shown a normal neuronal maturation in OHSC and an intact network integration of *in vitro* born granule cells (Gahwiler, 1984; Heimrich and Frotscher, 1991; Buchs et al., 1993; Raineteau et al., 2006; Chai et al., 2014).

Altogether, these observations demonstrate that OHSC do permit the survival and differentiation of granule cell progenitors *per se*. Therefore, we conclude that the apparent loss of eGFP-expressing progenitors in OHSC might be caused by an accelerated maturation of this cell population without an appropriate replenishment by the neurogenic niche. We hypothesize that processes exclusively taking place in OHSC might be responsible for the affected neurogenesis.

### Loss of Neurogenesis Markers Points to Disturbed Notch Signaling

To get insight into the cellular and molecular processes taking place in the neurogenic niche of OHSC, we analyzed the gene expression profile during cultivation. Hes5, which is a target gene of the Notch signaling cascade, exhibited a fast and strong decrease. Hes5 is a transcription factor centrally involved in stem cell maintenance and known to suppress neuronal differentiation (Bertrand et al., 2002; Ross et al., 2003). It was shown previously that the loss of Notch signaling is followed by an accelerated differentiation of neural progenitor cells and a depletion of the neurogenic niche (Imayoshi et al., 2010; Sibbe et al., 2012). Therefore, we assume that our observed loss of neurogenesis might be caused by a reduced Notch signaling, followed by the accelerated differentiation of neuronal progenitors and an exhaustion of the neurogenic niche. The constant expression levels of NeuroD1 support the unaffected or even accelerated maturation, as NeuroD1 effectively drives neuronal differentiation. *In vivo*, NeuroD1 is expressed by neuronal progenitors but not by mature, NeuN-positive granule cells (Gao et al., 2009; Guo et al., 2014). Therefore, the constant expression of NeuroD1 seen in our study is somehow surprising, as all other results point to a shrinkage of the progenitor cell pool. The stable NeuroD1 expression could have two possible causes: either NeuroD1 is not downregulated after granule cell maturation, or granule cells do not reach full maturity *in vitro*. A strong argument against the latter possibility is the fact that after 14–21 DIV most, if not all, OHSC granule cells were found to be NeuN-positive (data not shown) as well as the findings of other groups showing a normal maturation of granule cells in OHSC (Gahwiler, 1984; Heimrich and Frotscher, 1991; Buchs et al., 1993; Raineteau et al., 2006; Chai et al., 2014). It is known that the individual steps of neurogenesis are interconnected and that, for example, the reduction of one of the progenitor cell populations is followed by the proliferative activation of quiescent Type-1 stem cells (Seri et al., 2001; Ming and Song, 2011). However, this balance seems to be disturbed in OHSC, as the loss of neuronal progenitors is not accompanied by induced proliferation of stem cells.

### The Strong Activation of Glial Cells Seems to Underlie the Loss of Neurogenesis *In Vitro*

To enlighten potential reasons for the decreased neurogenesis, we studied neuroinflammatory processes taking place in OHSC, as it is well-known that strong inflammation can have deleterious effects on neurogenesis (Russo et al., 2011; Belarbi and Rosi, 2013). We found a fast and prominent pattern of astro- as well as microglial activation together with the upregulated expression of pro-inflammatory cytokines during OHSC cultivation. Although, it is widely believed that pro-inflammatory cytokines affect all steps of neurogenesis including progenitor cell differentiation (Sierra et al., 2014), there is evidence that IL-1β and IL-6 can also promote neuronal differentiation (Barkho et al., 2006). This could explain our observation of a non-restricted neuronal differentiation in OHSC despite strong inflammation.

By pharmacologically counteracting inflammatory processes with PSB 0739 and indomethacin, we were able to significantly protect the *in vitro* neurogenesis and to delay its decrease. The additional promotion of astroglial activation by treating OHSC with CNTF further accelerated the loss of neurogenesis and increased the expression of astroglial activation markers. Therefore, it is reasonable to assume a causal relationship between glia activation and the loss of neurogenesis in OHSC.

### The Role of Specialized Glial Cell Functions and Pro-inflammatory Cytokines

Pro-inflammatory cytokines are key candidates of mediating the anti-neurogenic effects of inflammation (Carpentier and Palmer, 2009; Kuzumaki et al., 2010; Wu et al., 2012; Belarbi and Rosi, 2013; Sierra et al., 2014). Conversely, we found that in OHSC anti-inflammatory treatments protected neurogenesis, but did not effectively decrease the expression of glial activation markers or of pro-inflammatory cytokines. Together with the observation that CNTF accelerated the neurogenic decline but slightly reduced IL-1β expression at all measured time points, we conclude that there have to be additional mechanisms interfering with neurogenesis besides pro-inflammatory cytokines. Accordingly, a recent study demonstrated that although Nestin-positive stem cells express IL-1 receptors, IL-1β acts anti-neurogenic in an indirect way without the recruitment of IL-1 signaling in stem cells (Wu et al., 2013).

There is recent evidence that under strong epileptic conditions neural stem cells start to divide symmetrically and subsequently transform into reactive astrocytes (Heinrich et al., 2006; Ledergerber et al., 2006; Sierra et al., 2015). Importantly, we were able to show that the generation and activation of astrocytes is not restricted to the DG but takes place throughout the whole OHSC, wherefore astrogliosis is likely to be a cause and not a consequence of the impaired neurogenesis. We observed that, after treatment with CNTF, OHSC showed an accelerated activation of astrocytes and, in parallel, lost their neurogenic capacity faster than under control conditions. In addition, indomethacin treatment, which non-selectively inhibits inflammation, was more effective in protecting neurogenesis and in stabilizing the Notch signaling than the microglia-specific PSB 0739 treatment. Altogether, these results point to a central role for astrocyte activation in the observed impairment of neurogenesis.

There is convincing evidence that, in contrast to astrocytes from other brain regions, hippocampal astrocytes are important for promoting neurogenesis in the SGZ (Song et al., 2002). They showed that astrocytes constitute an important part of the SGZ microenvironment, influencing the sequence of neurogenesis. Therefore, we hypothesize that under neuropathological conditions, like persistent inflammation, astrocytes might become activated in a way that they lose their neurogenesis-regulating capacity, followed by a depletion of the stem cell pool. One possible mediator could be the Notch signaling, as the expression of the Notch1 receptor as well as of its ligands by astrocytes, at least in the subventricular zone, was shown by Givogri et al. (2006). In this context, the strength and duration of inflammatory processes seem to determine the ultimate influence on the neurogenic niche and respective signaling pathways (Ekdahl et al., 2009; Belarbi and Rosi, 2013). Especially the chronification of inflammatory processes was shown to be detrimental for hippocampal neurogenesis (Ekdahl et al., 2003).

It is well-known that gliosis is a dynamic and sophisticated process and that glial cells highly interact in their cellular response. Therefore, under different neuropathological conditions microglia do not react in a binary way (active or inactive) but they rather exhibit a broad spectrum of activation states, which determines their diverse influence on neurogenesis (Butovsky et al., 2006; Walton et al., 2006; Ekdahl et al., 2009; Sierra et al., 2010; Graeber et al., 2011; Kettenmann et al., 2011; Belarbi and Rosi, 2013). This spectrum of activation states might therefore also apply to astrocytes, and it could be worthwhile to study the neurogenesis-supporting properties of astrocytes under varying inflammatory conditions.

## Conclusion

Our findings suggest that OHSC are a neuroinflammation model *per se*. This provides the opportunity to study the molecular basis of inflammatory conditions, which in OHSC do not seem to interfere with neuronal viability but instead cause a strong and specific reduction of neurogenesis. This knowledge is of high clinical relevance and a necessary prerequisite for developing strategies to control the microenvironment of the neurogenic niche in a way that new neurons can be generated even under pathological conditions.

## Author Contributions

CH: conception, supervision, manuscript writing; JG: performance of the experiments, data analysis, manuscript writing; CD: performance of the experiments; GM: performance of the experiments.

## Conflict of Interest Statement

The authors declare that the research was conducted in the absence of any commercial or financial relationships that could be construed as a potential conflict of interest.

## ABBREVIATIONS

BrdU: bromodeoxyuridine
CNTF: ciliary neurotrophic factor
DCX: doublecortin
DG: dentate gyrus
DIV: days *in vitro*
eGFP: enhanced green fluorescent protein
GFAP: glial fibrillary acidic protein
GCL: granule cell layer
H: hilus
Hes5: hairy and enhancer of split 5
Iba1: ionized calcium-binding adapter molecule 1
IL-1β: interleukin 1β
MHCIIAβ: major histocompatibility complex class II A β1
ML: molecular layer
NeuN: neuronal nuclei
OHSC: organotypic hippocampal slice cultures
PB: phosphate buffer
PCL: pyramidal cell layer
PFA: paraformaldehyde
POMC: proopiomelanocortin
SGZ: subgranular zone
SO: stratum oriens.
