# Supplementary figures and images for: Persistent Gliosis Interferes with Neurogenesis in Organotypic Hippocampal Slice Cultures
Source: Front Cell Neurosci. 2016 May 18;10:131. doi: 10.3389/fncel.2016.00131 (PMC4870256; doi:10.3389/fncel.2016.00131)

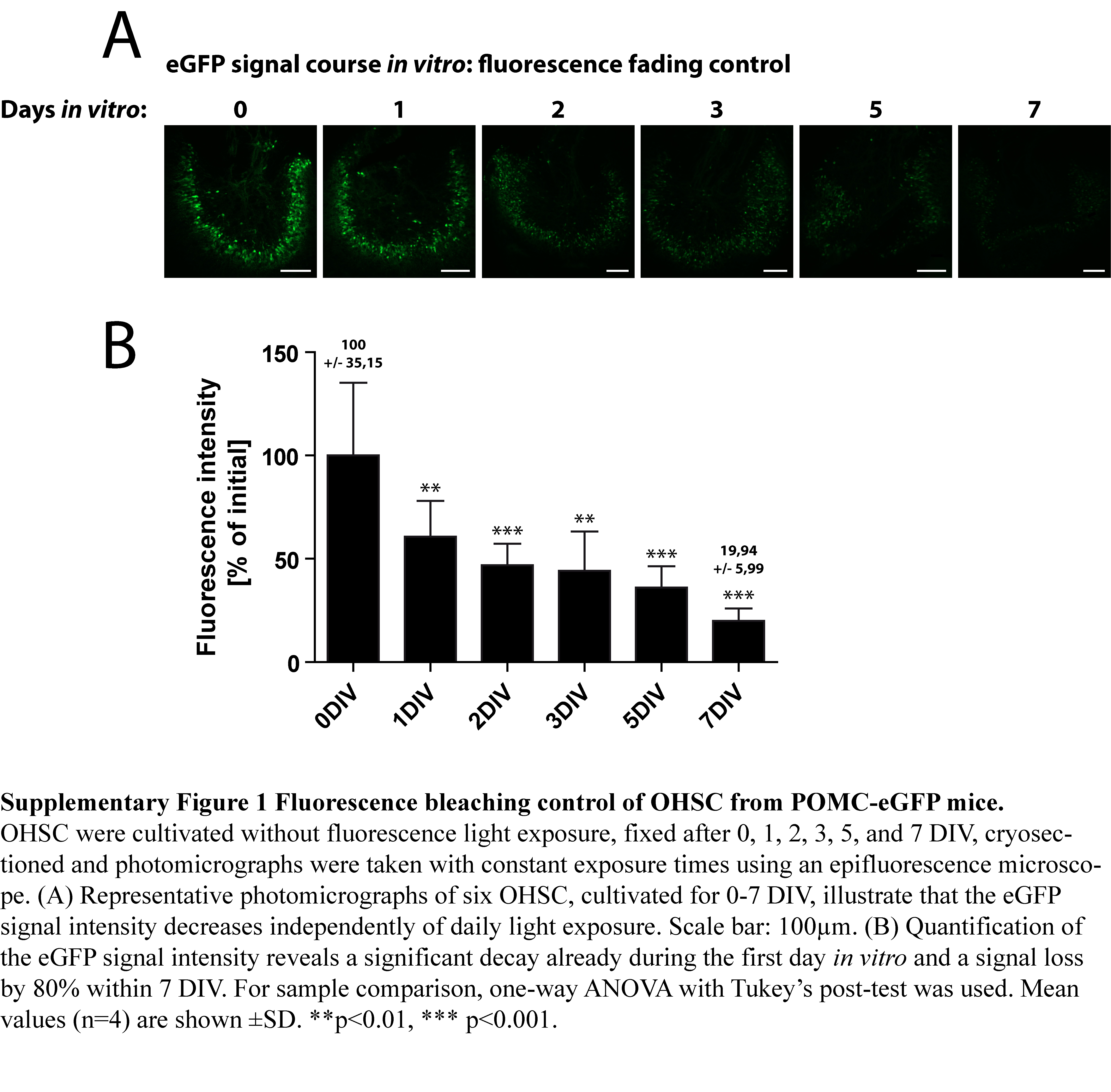

Supplement: Supplementary file 1 [file Image_1.TIF]

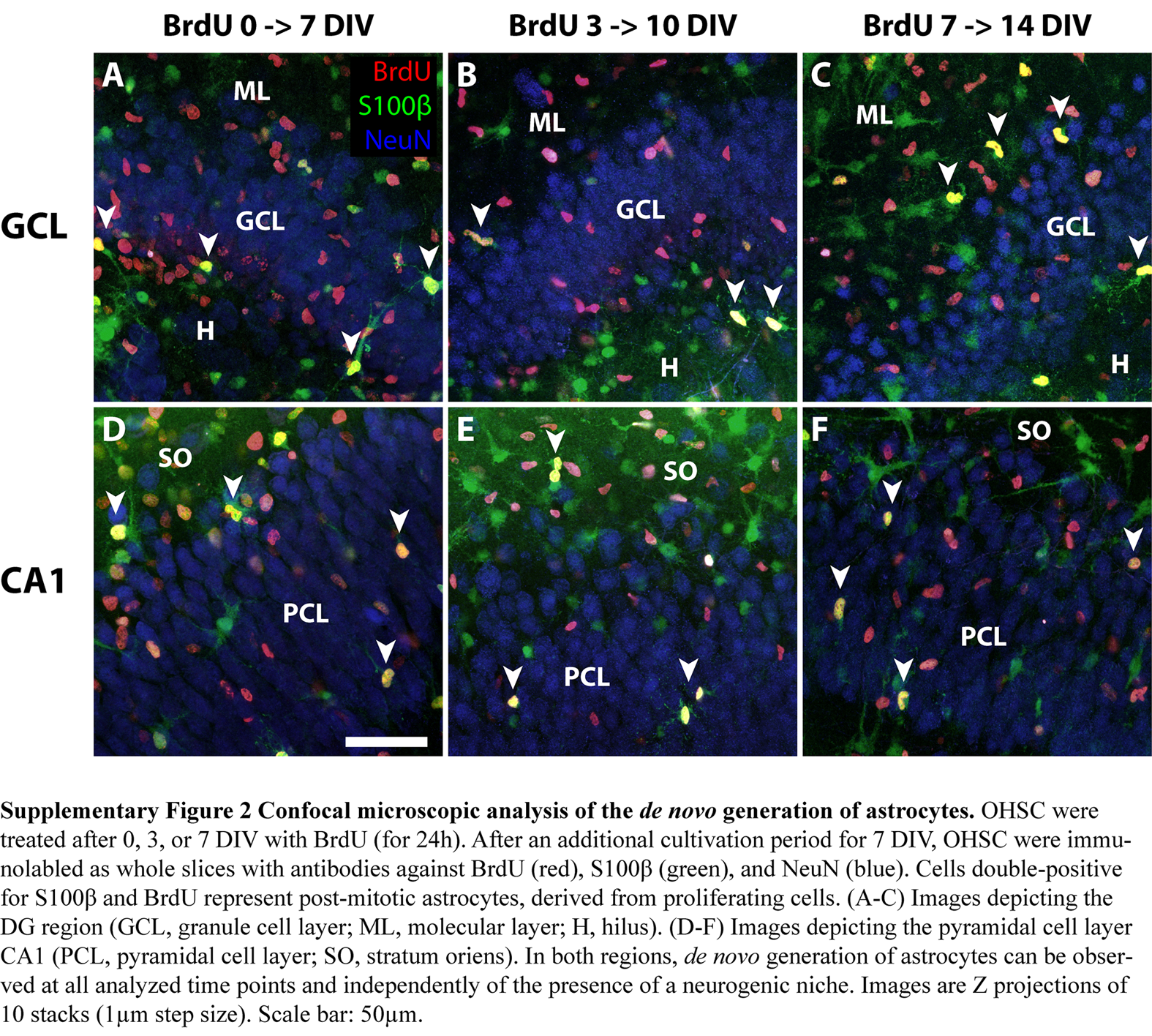

Supplement: Supplementary file 2 [file Image_2.TIF]
